# Unveiling Anxiety and Post-Traumatic Stress Disorder in Healthcare Workers Through Pre-Employment Evaluations

**DOI:** 10.1192/j.eurpsy.2026.10791

**Published:** 2026-07-31

**Authors:** N. Belhadj, N. Gannoun, R. Nakhli, C. Sridi, M. Bouhoula, M. Makhloufi, I. Kacem, A. Aloui, J. Ben Khelifa, F. Chelly, I. Fki, M. Maoua

**Affiliations:** 1 Occupational Medicine Department, Sahloul University Hospital; 2 Faculty of Medicine of Sousse, University of Sousse; 3 sousse, Research laboratory (LR19SP03); 4 Occupational Medicine Department, Farhat Hached University Hospital of Sousse; 5 Clinical Psychology Unit, Occupational Medicine Department, Sahloul University Hospital, sousse, Tunisia

## Abstract

**Introduction:**

Anxiety disorders and post-traumatic stress disorder (PTSD) are among the most prevalent mental health conditions worldwide, with healthcare professionals being particularly vulnerable due to their constant exposure to high-stress environments. Pre-employment psychiatric evaluations provide a critical opportunity to identify such conditions, assess their potential impact on professional functioning, and implement preventive strategies.

**Objectives:**

This study aimed to describe cases of anxiety disorders and PTSD identified during pre-employment medical assessments of healthcare workers.

**Methods:**

We conducted a descriptive cross-sectional study based on the medical records of healthcare workers assessed in our Department of Occupational Medicine during 2024. Each pre-employment evaluation included a clinical examination and a structured psychological interview exploring psychiatric history, current psychological state, and potential occupational implications.

**Results:**

A total of six healthcare workers were included (five women and one man), with a mean age of 31.5 years (range: 28–40). Reported conditions included specific phobias such as claustrophobia (1 case) and acrophobia (1 case), persistent stress and recurrent anxiety symptoms (2 cases), and post-traumatic stress disorder following bereavement or occupational exposure (2 cases). Among those with PTSD, one nurse required cognitive-behavioral therapy and grief management, and was declared fit for work with restrictions, specifically avoiding assignments in high-stress services such as emergency units. Another case required regular psychological follow-up. Despite the presence of these disorders, all participants were ultimately declared fit for work, with two cases requiring adapted recommendations or ongoing support.

**Conclusions:**

**Discussion/Conclusion:** The presence of anxiety disorders and PTSD among healthcare workers illustrates the psychological impact of high-stress medical environments. While these conditions were not a barrier to employment, they underline the importance of individualized assessment and workplace adjustments. Early screening and tailored support can help prevent relapse and maintain long-term professional performance.

**Disclosure of Interest:**

None Declared

